# Bilateral radial clubhand in a 6-year-old boy treated with ulnar radialization in a low-income country. A case report

**DOI:** 10.1016/j.ijscr.2025.111860

**Published:** 2025-08-21

**Authors:** Manitr'’Oliva Iarimanalina Ranaivoson, Thomas Daoulas, Pierre Maincourt, Rakotoherisoa Herijaona Manasse, Christophane Als Anselme Anesy, Malinirina Fanjalalaina Ralahy

**Affiliations:** a Pediatric Surgery Department, Andrainjato Fianarantsoa University Hospital, Madagascar; b Orthopedic Surgery Department, Cavale Blanche University Hospital, 29200 Brest, France; c Orthopedic Surgery Department, HJRA University Hospital, Antananarivo, Madagascar

**Keywords:** Deformity, Pediatrics, Radial club hand, Case report

## Abstract

**Introduction:**

Radial club hand is a rare congenital malformation with functional and aesthetic repercussions.

**Presentation of case:**

We present the case of a 6-year-old boy with bilateral hand deformity, associated with hypoplasia of the thenar muscles and aplasia of the radius (Bayne stage IV). After bilateral ulnar radialisation, recovery was favorable, with stability of the hand axis and a fine grip allowing writing. At 18 months postoperatively, the child was able to integrate into school without assistance.

**Discussion:**

Radial clubhands are rare malformations, often treated late in Madagascar due to a lack of resources. Despite these challenges, simple techniques like ulnar radialization can achieve excellent outcomes with minimal equipment.

**Conclusion:**

Surgery remains essential, even in cases treated late.

## Highlights

• **Radial club hand** is a congenital malformation that can lead to functional and aesthetic challenges, and surgical intervention, such as ulnar radialization, is crucial for improving hand function.
• In resource-limited settings, **ulnar radialization** proves to be a reliable and simple surgical solution, offering good functional outcomes with minimal complications.
• Postoperative recovery is typically uncomplicated, and in this case, the child achieved functional improvements.
• **Surgical treatment in low-income countries** must be optimized to minimize the need for multiple interventions, as patients often cannot afford follow-up surgeries or costly medical care.
• Effective surgical interventions like ulnar radialization can provide good long-term outcomes when access to specialized care is limited.

## 1 Introduction

Radial clubhand is a relatively rare congenital malformation with an annual incidence of 0.2 to 0.5 per 10,000 live births [1]. Characterized by a permanent deformity of the hand, deviated from the axis of the forearm. It results from radial aplasia and is associated with musculoskeletal and neuromuscular abnormalities on the radial side, sparing the ulnar structures [2]. Management varies from manual therapy to corrective surgeries.

In resource-limited settings, patients often present late, making early interventions like splinting impractical.

This case report has been reported in line with the SCARE 2025 criteria. [3].

## 2 Observation

The patient, a six-year-old boy, presented with bilateral congenital RCH, exhibiting a flexion-pronation deformity of the hand and an irreducible radial deviation. Hypotrophy of the thenar muscles and absence of the radial pulse were reported. The thumb, although hypoplastic (stage IIIA according to Blauth's classification), retained satisfactory motor function [4].

The functional QuickDASH scores before surgery were 72.40/100 on the right and 68.18/100 on the left. Radiographs revealed a type IV malformation according to the modified Bayne-Goldfarb classification, with complete absence of the radius, hypoplasia of the scaphoid, trapezium, and trapezoid. The hand-to-forearm angulation was 40° on the right and 45° on the left, according to the Manske index [5].

A bilateral ulnar radialization was performed. The first stage involved the release of the ulnar head and carpal bones after resection of the fibrosis.

**Fig. 1:**
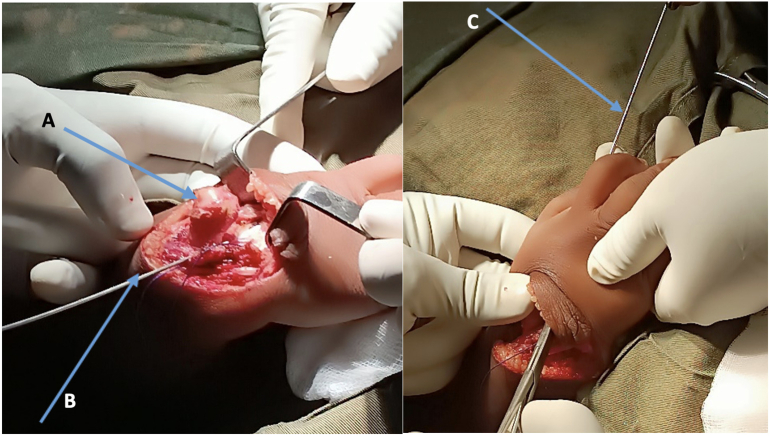
(A) Release of the ulnar head and carpal bones. (B) Insertion of a 1.5 mm threaded Kirschner wire through the carpal bones, exiting at the level of the second ray. (C) Radialization of the ulna maintained using the same wire with a back-and-forth technique.

**Fig. 2:**
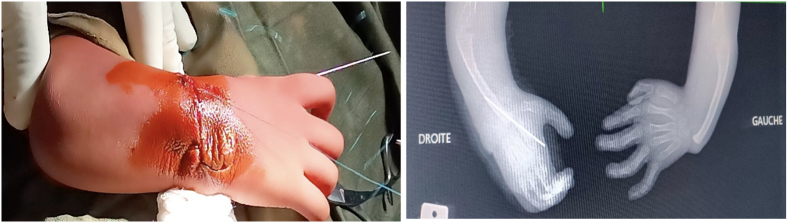
Immediate postoperative image demonstrating satisfactory correction and fixation, with the pin anchored in the posterior cortex of the ulna. No recoil of the ulna was observed after 3 months of follow-up on the right hand.

The second stage consisted of deformity correction, with the principle of repositioning the carpus onto the ulnar head, rather than repositioning the ulnar head onto the carpus, which would require shortening the ulna. *A*1.5 mm threaded Kirshner pin was placed in a “back-and-forth” manner, from the ulna to the second metacarpal (Fig. 1). The threaded wire enters the medullary cavity of the ulna. It must be fixed on the posterior ulnar cortex to prevent any recoil of the pin (Fig. 2).

The final stage involved reattaching the radial extensor tendon of the carpus to the ulnar osteo-aponeurotic elements using an osteosuture with slow-resorbing thread. The excess ulnar skin of the wrist was excised while preserving the sensory branches of the ulnar nerve.

**Fig. 3:**
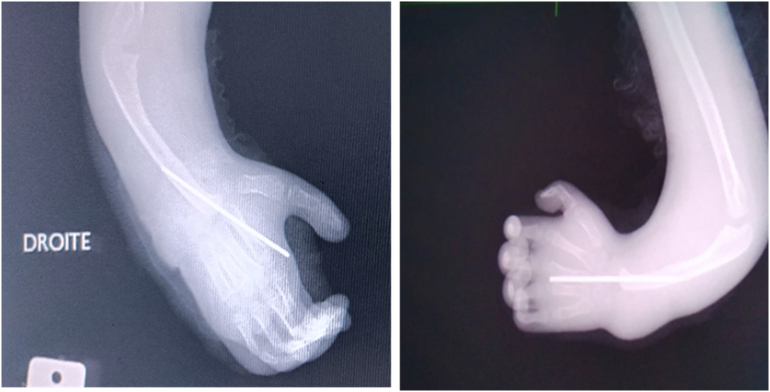
Follow-up radiographs at 3 months postoperatively, prior to pin removal, showing no secondary displacement or pin migration in either hand.

Postoperative recovery was uneventful, with postoperative Manske angles of 85° on the left and 80° on the right. The procedure was performed in two stages, separated by three months after removal of the pin on the right hand, to minimize perioperative disability and effectively manage postoperative pain. Skin healing was observed on day 10 on the right hand and on day 15 on the left due to clear fluid discharge from the surgical site, which quickly resolved with daily dressings. A posterior plaster cast (BABP type) was applied for 3 weeks and then replaced with an orthosis to maintain the correction. Rehabilitation and occupational therapy were initiated 3 weeks after the procedure, addressing joint mobility of the entire upper limb and focusing on grip strength and hand-to-mouth movement, allowing the patient to return to school 6 months after the last procedure. Hand-perineal reach was not possible during rehabilitation sessions, as the forearm was too short for this movement. The pins were removed 12 weeks after surgery; premature removal should be avoided due to the high risk of recurrence. (Fig. 3)

**Fig. 4:**
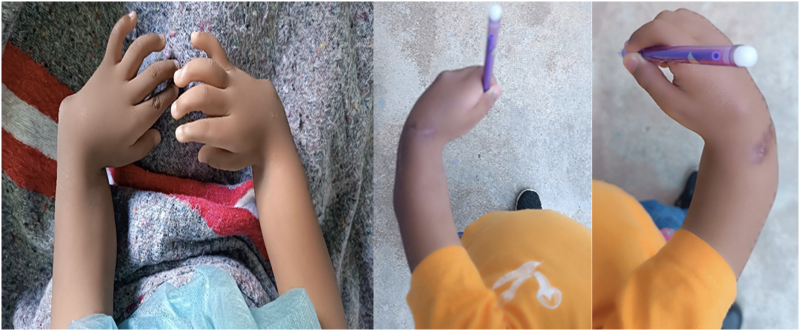
Clinical images of bilateral congenital radial club hand. Left: preoperative appearance; right: postoperative outcome at 18 months of follow-up.

At 18 months follow-up, the QuickDASH score was 15.91/100 for the right hand and 11.36/100 for the left hand (Fig. 4).

## 3 Discussion

Radial club hands (RCH) are rare congenital malformations, and their surgical management has not yet been fully standardized. However, therapeutic goals remain the same: cosmetic and functional.

In contexts such as Madagascar, where resources and working conditions are limited, patients often present at advanced stages of the condition due to the cost of care, rendering them ineligible for treatments involving splints or physiotherapy. Several studies recommend early-stage management using series splints in combination with manipulations performed by physiotherapists and parents to improve soft tissue flexibility before proceeding with surgical treatment around 12 months of age [6,7]. Result in minimal complications, as these patients cannot afford a second major surgery due to financial constraints.

Several surgical techniques have been described, including distraction methods using a mini external fixator and transfixing pins [8,9].

The choice of ulnar radialization was driven by its reliability and technical simplicity, not requiring expensive equipment. Additionally, this approach minimizes the need for multiple surgical interventions, aside from the pin removal. Postoperative recovery is typically uncomplicated and does not require the implementation of complex and costly postoperative protocols. The main risk associated with the radialization technique is recurrence, especially in cases of premature removal of the stabilizing pin and insufficient quality of the transferred muscles [7,10].

The intervention resulted in an excellent functional score. Good radio-clinical outcomes are particularly important given that the management of disability in adulthood remains extremely limited in Madagascar. Therefore, achieving optimal results from the first surgery is crucial, as these patients will not have access to further support. The stakes are even higher due to the significant out-of-pocket expenses for patients, many of whom cannot afford to finance costly medical care. In this context, evaluating the benefit-risk balance of each surgical intervention must be done optimally.

## 4 Conclusion

Ulnar radialization provides good outcomes, with simple and reliable recovery. This technique is well-suited for the management of radial club hands in low-income countries, where patients may face significant out-of-pocket expenses.

## CRediT authorship contribution statement

MOIR: Project administration; Resources.

HMR: Conceptualization.

PM: Data curation.

TD: Formal analysis; Funding acquisition.

AAAC: Supervision; Validation; Visualization.

RMF: Investigation; Methodology.

## Consent

Written informed consent was obtained from the patient's parents for publication and any accompanying images. A copy of the written consent is available for review by the Editor-in-Chief of this journal on request.

## Ethical approval

The local ethics committee approved the collection and publication of these data.

## Guarantor

Dr Thomas Daoulas: CHU de la Cavale Blanche, Boulevard Tanguy Prigent, 29200 Brest, France. Email: thomas.daoulas@outlook.fr. Phone: +33 6 36 82 88 07.

Dr Thomas Daoulas is the guarantor of this work and accepts full responsibility for the integrity of the data and the decision to publish.

## Declaration of Generative AI and AI-assisted technologies in the writing process

The authors affirm that no generative artificial intelligence (AI) tools were used in the writing, editing, or content generation of this manuscript. All text was written and revised solely by the authors themselves, based on their clinical expertise, analysis, and interpretation of the case.

## Sources of funding

None.

## Declaration of competing interest

The authors declare no conflict of interest.
